# Tryptophanyl tRNA synthetase is an alternative synovial biomarker for diagnosis of septic arthritis in knee joint

**DOI:** 10.1186/s43019-024-00229-2

**Published:** 2024-09-16

**Authors:** Byung Hoon Lee, Young Gon Na, Seong Hyup Ham, Mirim Jin, Yoon Tae Kim, Kyung-Ok Kim, Jae Ang Sim

**Affiliations:** 1 Department of Orthopaedic Surgery, Gil Medical Center, Gachon University College of Medicine, Incheon, 21565 Republic of Korea; 2 Department of Microbiology, College of Medicine, Gachon University, Incheon, Korea; 3 Department of Health Science and Technology, GAIHST, Gachon University, Incheon, Korea; 4 Gil Medical Center, Gachon Medical Research Institute, Gachon University, Incheon, Republic of Korea

**Keywords:** Septic arthritis, Biomarker, Tryptophanyl-tRNA synthetase, Knee

## Abstract

**Background:**

To evaluate the diagnostic characteristics of tryptophanyl tRNA synthetase (WRS) for the diagnosis of septic arthritis of the knee joint and to determine whether it is a reliable and sensitive synovial biomarker for discriminating septic arthritis from other types of arthritis.

**Methods:**

Patients joint effusions for which septic arthritis was suspected were prospectively recruited between January 2019 and September 2020. A total of 9 patients had septic arthritis, 6 had acute gout attack, 1 had an acute flare of chronic rheumatic arthritis, and 46 had pseudogout or reactive arthropathy. Traditional inflammatory markers were measured, and their diagnostic abilities were compared. Neutrophil count, C-reactive protein (CRP) level, WRS, and human neutrophil α-defensin levels were assessed in the synovial fluids. Demographic parameters and biomarkers with a *P* < 0.05 in differentiating septic from nonseptic arthritis were included in a multivariable model. A multivariable logistic regression with a stepwise selection was performed to build the final combined model. Receiver operating characteristic curves were used to establish optimal thresholds for the diagnosis of septic arthritis of the knee joint, and the area under the curve was calculated to determine the overall accuracy of these tests compared with patients with nonseptic inflammatory arthritis.

**Results:**

Patients with septic arthritis were more likely to display higher serum WBC and CRP levels, synovial neutrophil counts, and levels of two synovial biomarkers, including WRS and α-defensin. WRS showed the highest specificity (87.5%) and sensitivity (83.3%) with α-defensin among the three synovial biomarkers.

**Conclusions:**

Synovial fluid WRS is a relevant biomarker in discriminating septic arthritis from other inflammatory arthritis and should be tested in an independent cohort.

Level of evidence: prospective observational study, III.

## Background

If severe acute pain and swelling without any special traumatic history is accompanied by intra-articular effusion, purulent arthritis should be differentiated between bacterial infection or noninfectious, inflammatory arthritis. Septic arthritis represents a severe medical condition marked by the invasion of purulent exudate into the joint cavity, precipitated by an infectious pathogen. In the absence of timely medical intervention, septic arthritis can cause irreversible structural damage to the affected joints [1]. Antibiotic treatment is necessary after surgical treatment as soon as possible, and the delay in diagnosis and treatment accelerates damage to normal articular cartilage; thus, an early diagnosis is of paramount importance.

However, these two categories are not clearly distinguished, and as a result, unnecessary surgical treatment is often performed in inflammatory arthritis. In contrast, surgical treatment can be delayed, despite a septic arthritis diagnosis [2]. The diagnosis of septic arthritis was previously confirmed if bacteria could be cultured from inflammatory synovial fluid and/or blood samples. However, bacterial culture is not feasible in the emergency room. Therefore, in current clinical practice, laboratory tests, such as leukocyte count and neutrophil fraction (%), are performed using the joint fluid collected via articular puncture, and the diagnosis of septic arthritis relies on the assessment of clinical symptoms and blood biomarkers, including erythrocyte sedimentation rate (ESR) and C-reactive protein (CRP) [3–5]. The ideal thresholds and accuracy rates for tests detecting joint infections have fluctuated in literature, resulting in a lack of a clear, definitive method that can be used by orthopedic surgeons to diagnose septic arthritis [6–8].

Currently, there is interest in new types of synovial fluid biomarkers for the diagnosis of septic arthritis. A biomarker can be defined as a biochemical measure that is indicative of a biological process or the response to an intervention. Numerous biomarkers have been developed and have become available [9–11], including synovial leukocyte esterase [12–14], synovial alpha defensin, and synovial CRP [15–17], specifically for the diagnosis of septic arthritis, and they provide a rapid and accurate diagnosis [18].

A recent interest in a metabolomics approach has also been growing, as the metabolome is the result of gene and protein functions and activities [19–24], and new alternative biomarkers directly linked to specific conditions, such as osteoarthritis [25], have been suggested. Therefore, to treat septic arthritis in a clinical setting, it might also be advisable to identify the clinically available products for targeted metabolomics and to achieve an absolute quantification of defined metabolite classes. Tryptophanyl tRNA synthetase (WRS) is an essential enzyme, as it catalyzes the ligation of tryptophan to its cognate tRNA during translation [26]. WRS plays unique roles in physiological homeostasis and immune defense, and its responses eliminate invading pathogens in a very early phase of infection [27, 28].

This study aims to prospectively assess the diagnostic properties of WRS and to determine whether it is a reliable and sensitive synovial fluid metabolic marker for discriminating septic from other types of inflammatory arthritis. To determine the clinical applicability of WRS, its diagnostic accuracy was analyzed compared with α-defensin, which has been reported as the best synovial biomarker for the diagnosis of periprosthetic joint infection among several others [11].

## Materials and methods

### Study design

A total of 62 knees from 62 patients, who presented with acute monoarthritis and exhibited inflammatory synovial fluid (defined as a white blood cell count > 2000/mm^3^ and > 80% neutrophils [29]), indicative of septic arthritis, underwent prospective evaluation and subsequent revision by two fellow-ship trained lower extremity surgeons (B.H.L. and Y.G.N.) from January 2019 to September 2020. All patients were divided into two subgroups, one comprising 9 joints with septic arthritis and another comprising 53 joints with nonseptic inflammatory arthritis. When microbial culture tests were conducted on synovial fluid and/or blood samples, a diagnosis of septic arthritis was made if bacteria were identified and there were no crystals present. Additionally, microbial etiology was determined through Gram staining and culture test in the bacteriology laboratory. Genus and species identification were performed using biochemical assays (analytical profile index). The diagnosis of pseudogout was confirmed upon the observation of calcium pyrophosphate crystals in inflammatory and aseptic synovial fluid [30, 31]. A diagnosis of rheumatoid arthritis (RA) was confirmed based on the assessment of rheumatologists and the fulfillment of the respective American College of Rheumatology criteria for the conditions [32]. Institutional review board approval (GBIRB2017-330) was obtained from our institution (Gachon University Gil Hospital, Incheon, South Korea) before the study, and the protocol was approved. All patients provided informed consent prior to participation in this study.

All patients underwent serum ESR and CRP testing during the initial evaluation and joint aspiration was performed either before or during the operation. WBC count and a differential culture of synovial fluid were obtained. We analyzed and compared data from patients with inflammatory and noninflammatory arthritis, separately.

### Synovial fluid and biomarker analysis

Serum levels of ESR and CRP are measured from a venous blood sample obtained from each patient. Blood CRP levels were determined using nephelometry. A synovial fluid specimen was also collected for Gram staining, bacterial culture, white blood cell (WBC), and polymorphonuclear leukocyte counts. The collected synovial fluid samples were promptly transported to the laboratory and subsequently centrifuged at 2000 rpm for 20 min at room temperature to eliminate debris from the samples. All samples were immediately divided into 100 µL aliquots and frozen at −80 °C until analyses were conducted. Synovial biomarkers and α-defensin, WRS, and CRP levels were assessed in synovial fluids. α-defensin and WRS were evaluated using commercial ELISA kits (R&D Systems, Minneapolis, MN) according to the manufacturers’ instructions.

The multiplex assay and ELISA assay proceeded in a very similar manner. A multiplex assay was used to quantify α-defensin, WRS, and CRP levels in the synovial fluids. We added 25 µL of each sample and 25 µL of beads into individual wells of a 96-well plate, followed by overnight incubation at 4 °C. The following day, the plate contents were aspirated, and 25 µL of detection antibodies (targeting the analytes of interest) were dispensed into each well. The plates were left to incubate for 1 h at room temperature. Subsequently, 25 µL of phycoerythrin (PE)-conjugated streptavidin was added to each well containing the detection antibodies, along with 150 µL of sheath fluid. Beads were examined using a dual-laser system for bead classification and identification of the detected analyte. The second laser quantified the signal derived from PE, directly correlating with the quantity of bound analyte. Finally, we quantified the cytokine concentrations in the plates utilizing a cytometer (Luminex 200; Luminex, Austin, TX).

We used P211801 (α-defensin) and P225864 (CRP) kits for the ELISA assays from R&D Systems, and used the E-WA-1 (WARS1) kit from Biocon (Incheon, Korea). We dispensed 100 µL of assay diluent into each well. Following the removal of the assay diluent, 50 µL of samples were added in duplicate, and the plate was then incubated for a minimum of 2 h at room temperature. For antibody binding, we added 200 µL of human α-defensin/CRP conjugate and WARS antibody to each well, followed by incubation for 2 h at room temperature. The subsequent step involved adding 200 µL of substrate solution and allowing it to incubate at room temperature to facilitate appropriate color development. To terminate the color reaction, 50 µL of the stop solution were added. We used a microtiter plate reader (Molecular Devices, Emax) to measure the absorbance at 450 nm.

### Statistical analysis

An a priori power analysis was performed to determine an optimal sample size using a two-sided hypothesis test at a significance level of 0.05 and a power of 0.8. A post hoc power analysis was performed to determine whether adequate power (0.91–0.99) is indicated for the results of nine cases to detect a significant difference of measurement outcomes of Neutrophil percentages and the absolute leukocyte count (/mm^3^) between those of septic arthritis and nonseptic inflammatory arthritis in the present study [33]. The statistical analysis was performed using SPSS software (SPSS for Windows Release version 17.0; SPSS, Chicago, IL), significance being determined at the 95% level.

The results of all synovial fluid tests were examined and compared between cases with infection and those without. Signal-to-cutoff ratio was 1.0 to analyzing α-defensin assay, determined from a preliminary study, as the diagnostic threshold. The sensitivity and specificity of the α-defensin assay were calculated, along with their corresponding 95% confidence intervals (CIs). Within each patient group, the diagnostic performance of CRP, synovial fluid WBC count, and synovial fluid biomarkers for identifying joint infection was assessed using receiver operating characteristic curves. Test sensitivity was plotted against specificity for each threshold tested, and the area under the curve (AUC) was determined. Using Youden’s *J* statistic, optimal cutoff values corresponding to the cultured diagnosis were identified. Sensitivity, specificity, positive predictive value (PPV), and negative predictive value (NPV) at the chosen cutoff points were derived from model-based estimates, along with the corresponding 95% confidence intervals for these parameters. Using ROC curve analysis, the threshold for the biomarkers was established. Sensitivity, specificity, positive predictive value (PPV), and negative predictive value (NPV) were calculated at a predetermined threshold. Biomarkers with a *P* < 0.05 were incorporated into the multivariate model. Multivariate logistic regression with stepwise selection was performed to build the final combined model.

## Results

A total of 62 patients were included: septic arthritis (*n* = 9), pseudogout or reactive arthropathy (*n* = 46), gout (*n* = 6), and rheumatoid arthritis (*n* = 1). Nine patients diagnosed with septic arthritis had positive synovial fluid cultures. The identified bacteria were *Streptococcus sp*. in three patients (*pyogenes*, *dysgalactiae*), *Staphylococcus sp*. in four patients (*aureus*), *Escherichia coli* in two patients (Table 1). All patients were considered immunocompetent. Rheumatoid arthritis (RA) or pseudogout occurred in the native joints. The serum CRP level (20.60 ± 5.70 versus 9.50 ± 6.09, *P* < 0.001) and synovial fluid neutrophil count (142,778 ± 116,744 vs. 48,351 ± 34,370, *P* = 0.024) were both higher in patients with septic arthritis (Table 2). The serum CRP and synovial fluid neutrophil count were found to be highly sensitive tests in detecting joint infection, as their values are rarely within the normal range in the presence of infection. Among the three synovial biomarkers, CRP, WRS, and α-defensin were significantly higher in patients with septic arthritis than with nonseptic inflammatory arthritis, whereas the synovial CRP level did not show significant differences in between both groups (*P* = 0.359).

**Table 1:** Bacterial causes identified using joint fluid culture*

Bacteria	Septic arthritis group (*n* = 9)
Staphylococcal species	
Methicillin-sensitive *Staphylococcus aureus*	3 (33.3%)
Methicillin-resistant *Staphylococcus aureus*	1 (8.3%)
* Escherichia coli*	2 (16.7%)
Streptococcus species	
* Streptococcus pyogenes*	1 (25%)
* Streptococcus dysgalactiae*	2 (16.7%)

^*^The values are given as the number of infections in each group, with the percentage (total number of infections) in parentheses

**Table 2:** Comparison of demographic data and serum and synovial fluid analysis between septic and nonseptic inflammatory arthritis**

	Septic arthritis group (*n* = 9)		Nonseptic arthritis group (*n* = 53)		*P* value
	Mean	(Standard deviation)	Mean	(Standard deviation)	
Age (years)	72.67	(16.01)	68.87	(15.42)	0.522
Sex, Male [% (M:F)]	44.4% (4:5)		43.4% (23:30)		1.000*
Comorbidity, diabetes	33.3% (3/9)		22.6% (12/53)		0.434*
Body temperature (°C)	37.03	(0.47)	37.40	(1.03)	0.488
Blood tests					
Hb	10.73	(1.40)	11.31	(2.24)	0.392
WBC	13.70	(3.87)	9.85	(3.96)	**0.029**
Platelets	181.29	(46.72)	260.67	(100.12)	**0.049**
Diff. count of segmental neutrophils (%)	85.16	(8.90)	71.55	(11.86)	**0.006**
ESR	52.00		39.20	(33.24)	0.743
CRP	20.60	(5.70)	9.50	(6.99)	** < 0.001**
Uric acid	4.43	(0.67)	5.33	(2.04)	0.243
Rheumatoid factor			7.25	(2.19)	
Anti-CCP Ab^a^			5.60		
PT	12.30		11.42	(1.71)	0.652
PT sec	20.70	(9.76)	12.90	(2.02)	0.460
INR	1.60	(0.74)	1.12	(0.15)	0.384
PT percent	50.55	(31.47)	84.27	(19.68)	0.360
Synovial analysis					
Specific gravity	1.02	(0.01)	1.02	(0.01)	0.420
PH	7.75	(0.46)	7.81	(0.53)	0.740
RBC	13119.67	(4466.31)	8939.19	(18730.52)	0.282
WBC	142,778.00	(116,744.85)	48,351.46	(34,370.59)	**0.024**
Synovial biomarkers					
WRS (ng/mL)	773.43	(513.51)	159.17	(191.99)	**0.012**
α-defensin (ng/mL)	282.11	(71.69)	186.05	(132.83)	**0.013**
CRP^b^	830.74	(209.88)	900.20	(113.60)	0.359

Value of *P* < 0.05 are displayed in bold

^*^Dichotomous variables are presented as ratios (percentage), and continuous data are presented as mean and standard deviation. The Wilcoxon rank sum test or Fisher’s test (septic arthritis versus nonseptic arthritis) were used when appropriate

^a^*CCP Ab* Cyclic citrullinated peptide antibody

^b^*CRP* C-reactive protein

In the multivariate model, which included the blood CRP level, synovial fluid α-defensin, and WRS level, WRS was the most discriminating diagnostic biomarker, distinguishing septic arthritis from nonseptic inflammatory arthritis with an 88.9% sensitivity, an 88.7% specificity, a 57.1% PPV, and an 88.7% NPV. The optimal diagnostic threshold of serum CRP and synovial fluid WBC count were 18 mg/L and 81,120/µL, respectively, for patients with septic arthritis. Similarly, the optimal threshold for the synovial fluid WBC count was 81,120/µL for patients with septic inflammatory arthritis. Finally, the optimal thresholds for the synovial fluid biomarkers of WRS and α-defensin were 418 and 301, respectively (Table 3).

**Table 3:** Optimal diagnostic thresholds and their sensitivities, specificities, and predictive values for predicting joint infection

Test	Optimal diagnostic threshold	Sensitivity (%)	Specificity (%)	Positive predictive value (%)	Negative predictive value (%)	Area under receiver operating characteristic curve (%)	*P* value for area under curve
Serum CRP (mg/L)	18.210	77.7	49.1	20.5	92.8	86.5	**0.010**
Synovial fluid							
WBC count (/µL)	81.120	77.7	92.4	63.6	96.0	91.7	**0.003**
WRS (ng/mL)	417.9	88.9	88.7	57.1	88.7	88.5	**0.008**
α-defensin (ng/mL)	300.8	88.9	86.8	53.3	97.8	82.3	**0.022**
CRP	950.0	55.5	54.7	17.2	87.8	54.7	0.740

Boldface text indicates statistical significance

**Fig. 1:**
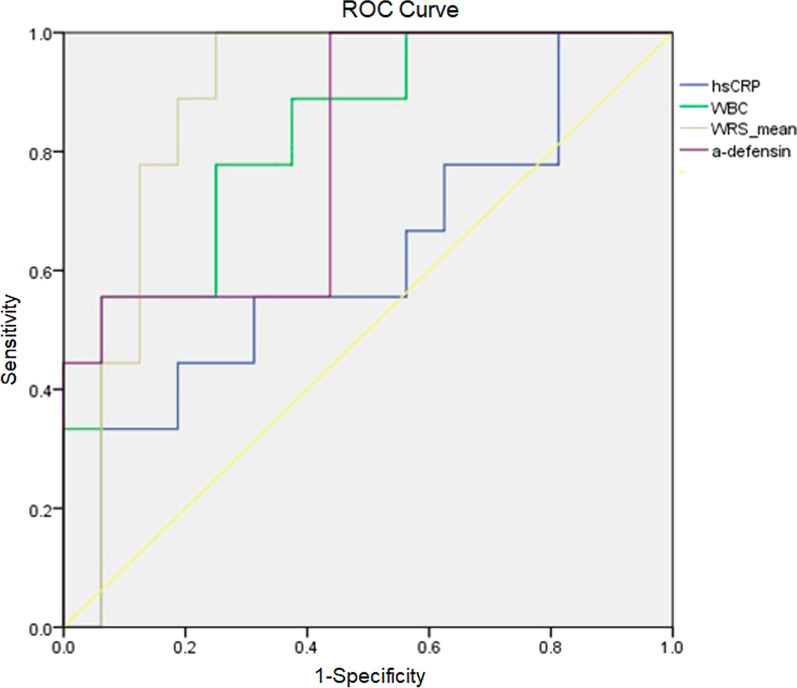
Receiver operating characteristic curves with the calculated area under the curve (AUC) comparing patients with septic and nonseptic inflammatory arthritis. Cutoff values that were selected to maximize the sensitivity and specificity are indicated on each curve. *hsCRP* human serum CRP, *WBC* synovial fluid WBC count, *WRS* synovial fluid WRS, *α-defensin* synovial fluid α-defensin

Figure 1 illustrates the receiver operating characteristic curves for the three synovial biomarkers and their differentials in each patient population. The sensitivities, specificities, PPV, and NPV for the blood CRP level, synovial fluid WBC count, α-defensin, and WRS levels were comparable for patients with septic and nonseptic inflammatory arthritis. Furthermore, the comprehensive accuracies of each assessment, delineated by the area under the curve, demonstrated significant efficacy. For the population of patients with septic arthritis, the synovial fluid WRS was the most accurate biomarker.

## Discussion

Our results demonstrated that WRS could be an alternative synovial biomarker for the diagnosis of septic arthritis of the knee joint with high sensitivity and specificity, even compared with the synovial CRP level, synovial fluid WBC count, and α-defensin.

There has been a notable change in the paradigm concerning the ideal candidate for aspiration [34, 35], the appropriate assays to conduct on aspirated fluid [36, 37], and the significance of aspiration in establishing the presence or absence of septic arthritis [30, 38, 39]. Recent research concerning biomarkers for septic arthritis has primarily centered on prosthetic joint infection, while information regarding infection in native joints is limited [40–42]. However, distinguishing between inflammatory arthritis during a flare-up or crystal-induced arthritis from septic arthritis poses additional challenges in managing arthritis among the elderly [43]. The risk of septic arthritis increases with comorbidities [44]. In this study, there was also a higher rate of diabetes in the septic arthritis group.

Gram staining and culture methods are extensively employed for the detection and characterization of infectious agents in blood and various bodily fluids. Many authors recommend Gram staining and culturing of synovial fluid in cases where septic arthritis is suspected to detect and identify infectious agents [45, 46]. The specificity of culture was estimated to be over 90% and “quite high” for Gram stain [1], but it still varied over time in literature. Only about 50% of the actual purulent arthritis is identified using the bacterial culture test, and in many cases, it is inevitable to decide the surgical treatment based on clinical judgment and on each synovial and blood test. Hence, the leukocyte count in synovial fluid and the assessment of PMN% are considered reliable tests for patients with both inflammatory and noninflammatory arthritis. Nevertheless, the threshold values for leukocyte count and PMN% exhibit some variability, potentially impacting the precision of the tests [36, 47–49].

Lately, there has been a conspicuous increase in the importance of metabolomics in the exploration of disease biomarkers and metabolic pathways [50, 51]. Several investigators have sought to identify reliable biomarkers for this specific disease spectrum in the serum, synovial fluid, and other body fluids. Metabolites denote both intermediate and final products resulting from diverse cellular processes, with their concentrations being a consequence of the response of biological systems to genotypic and environmental influences [25]. These biomarkers consist of inflammatory proteins, such as cytokines, which are vital in the host’s response to pathogens, including antimicrobial peptides [52]. However, as with other biochemical measures, it is difficult to interpret these synovial fluid biomarker [53], and there are currently no pertinent biomarkers accessible for diagnosing this disabling condition.

α-defensin is an antimicrobial peptide released by human neutrophils in reaction to the presence of pathogens [54, 55]. α-defensin plays a role in depolarizing the cell membrane of pathogens, leading to their destruction. α-defensin can be identified by laboratory-based α-defensin enzyme-linked immunosorbent assay (ELISA) or by employng an α-defensin test kit. Several studies have indicated that α-defensin in synovial fluid exhibits the highest potential as a biomarker in terms of sensitivity and specificity for diagnosing periprosthetic joint infection [9, 12, 15, 56], followed by IL-8 [11]. Our results also reinforced the clinical applicability of α-defensin with a high accuracy to distinguish septic arthritis from other inflammatory arthritis types.

CRP, another synovial biomarker, is a prominent inflammation marker and has long been utilized as an indicator of infection within the field of orthopedic surgery. Like α-defensin, the release of CRP is triggered by the recognition of pathogenic patterns, contributing to various mechanistic functions within the innate immune response. In certain studies, synovial CRP has been deemed more effective than serum CRP as a biomarker [57–59]. However, Tetreault et al. [17] found that synovial and serum CRP levels exhibited comparable sensitivity, specificity, and accuracy. Our data on native joint infection showed no statistically significant differences between septic and non-septic inflammatory arthritis.

Tryptophanyl tRNA synthetase (WRS) is an essential enzyme, as it catalyzes the ligation of tryptophan to its cognate tRNA during translation [26]. WRS can also further expand its functions via alternative splicing and proteolytic cleavage. WRS is localized not only in the nucleus but also in the extracellular space, playing a key role in innate immunity, angiogenesis, and IFN-γ signaling. WRS is secreted into the extracellular space in response to certain stimuli. For example, upon pathogenic infection but prior to tumor necrosis factor-α (TNF-α) production, WRS is rapidly secreted from monocytes without de novo synthesis; although, the mechanism of secretion is still not completely known [26]. In addition, the expression of WRS varies significantly in different tissues and pathological states, implying that it plays unique roles in physiological homeostasis and in immune defense. The secreted full-length (FL)-human WRS (FL-WRS) leads to the activation of innate immune responses, in which TNF-α and chemokine production, neutrophil infiltration, and increased phagocytosis are prominent. These responses eliminate invading pathogens in the very early phase of infection, implying that there is a crucial role of FL-WRS in countering infections and immune regulation [27, 28]. The laboratory-based ELISA test demonstrated that synovial fluid WRS showed the highest sensitivity and specificity for discriminating septic arthritis from other inflammatory arthritis, compared with other relevant synovial biomarkers. The sensitivity, specificity, negative predictive value, and positive predictive value for all tests were also comparable in both groups. To the best of our knowledge, this is the first study to use a metabolomics approach to classify patients with septic arthritis in inflamed knee joints and the first report on the use of WRS in discerning septic arthritis from inflammatory joint disease.

We aim to ascertain the most effective cutoff values for these assessments, considering potential variations between patients with a history of inflammatory and noninflammatory arthropathy. The standard cutoff value of synovial fluid leukocytosis to rule out septic arthritis is recommended to be 50,000/mm^3^. However, several researchers have suggested that the count lacks the sensitivity required for clinical utility in excluding infectious arthritis [8]. Our results showed that the optical cutoff synovial fluid WBC count to distinguish septic arthritis was 98,736/mm^3^. In addition, the optical cutoff level of serum CRP was 18.2 mg/L.

The current study has some limitations. First, in this study, the confirmation of septic arthritis diagnosis relied solely on the presence of bacteria in cultures obtained from synovial fluid and/or blood samples. Although the specificity of synovial culture bacteriology has been reported to be over 90%, some studies have reported a variable positive rate of 67% [1]. However, we still think that culture bacteriology is the only definitive diagnosis method of septic arthritis. Synovial biomarkers have demonstrated promising outcomes, suggesting their utilization as diagnostic supplements alongside synovial white cell count and bacterial culture analysis. Limitations of this study involve the relatively small cohort size, particularly when compared with the number of outcomes measured. Nonetheless, to address this limitation, synovial biomarker levels were measured using the multiplex assay and ELISA to achieve a high accuracy. The multiplex assay uses a procedure very similar to that of the ELISA assay but has the merit of being able to detect up to 100 or more factors at once using a small amount of sample. The ELISA assay is one of the most widely used techniques because it is simple and accurate, being able to analyze many samples at once. The multiplex assay has the merit of saving reagents and consumables, quickly detecting multiple factors at the same time, and efficiently using valuable samples, measured simultaneously, only a small amount of sample is necessary, and it has a high accuracy and sensitivity [60–62]. Nevertheless, each individual test comes with a substantial commercial cost, which serves as a restricting factor. Cost-effectiveness analyses will ascertain whether the expenses associated with this new tool are warranted.

## Conclusion

Synovial fluid WRS is a relevant biomarker to discriminate septic arthritis from other inflammatory arthritis and should be tested in an independent cohort.

## Data Availability

The datasets used and/or analyzed during the current study are available from the corresponding author upon reasonable request.
